# Alternative activation

**DOI:** 10.7554/eLife.89112

**Published:** 2023-06-12

**Authors:** Kristen Young, Sean Fanning

**Affiliations:** 1 Stritch School of Medicine, Loyola University Chicago Maywood United States

**Keywords:** nuclear receptors, NMR spectroscopy, ligand binding, transcription factors, biophysics, protein-protein interaction, None

## Abstract

A detailed study of the orphan receptor Nurr1, a regulator implicated in neurodegenerative diseases, reveals a new way for ligands to control their transcriptional activity.

**Related research article** Yu X, Shang J, Kojetin DJ. 2023. Molecular basis of ligand-dependent Nurr1-RXRα activation. *eLife* **12**:e85039. doi: 10.7554/eLife.85039.

Between 10 to 20% of all FDA-approved drugs target a single class of proteins that is critical to human development and physiology across all tissues (Weikum et al., 2018; Dhiman et al., 2018). Known as nuclear receptors, these master regulators can attach to DNA to coordinate transcriptional programs that modify cellular fitness or function (Nettles and Greene, 2005). They are often activated when specific ligands such as metabolites or hormones directly bind onto them at dedicated sites or ‘pockets’ (Olefsky, 2001).

Many nuclear receptors, and in particular many ‘orphan’ nuclear receptors for which an endogenous ligand has yet to be identified, are also involved in disease (de Vera, 2018). Estrogen receptors, for instance, can alter the transcription of thousands of genes in breast cancer cells (Frasor et al., 2003).

Another example is Nurr1, an orphan nuclear receptor critical for the development and maintenance of the neurons that produce dopamine (Zetterström et al., 1997). This receptor has been implicated in dementia, Alzheimer’s and Parkinson’s disease, as well as other neurodegenerative disorders (Jeon et al., 2020; Chu et al., 2002; Decressac et al., 2013). As the expression of Nurr1 diminishes with age, reactivating its production has potential as a therapy against these conditions (Moutinho et al., 2019). Yet designing small molecules that specifically target Nurr1 has been difficult so far, as the canonical ‘pocket’ which normally welcomes ligands is absent on this receptor (Wang et al., 2003).

An alternative approach may be to target RXRα, a nuclear retinoid receptor which has also been highlighted as a drug target for Alzheimer’s and Parkinson’s diseases. Nurr1 and RXRα bind together to form heterodimers that result in Nurr1 transcriptional activity being repressed (Aarnisalo et al., 2002; Cramer et al., 2012; Friling et al., 2009). In turn, several RXRα ligands and targeted small molecules can modulate the activity of Nurr1, but exactly how this phenomenon takes place remained unclear (Scheepstra et al., 2017). Now, in eLife, Xiaoyu Yu, Jinsai Shang and Douglas Kojetin, who are based at Scripps Research, report using a comprehensive suite of biophysical and structural approaches to reveal how RXRα ligands promote the transcriptional activation of Nurr1 (Yu et al., 2023).

First, the team used reporter gene assays to examine how RXRα as well as various ligands affect Nurr1 transcription in neuronal cells. The experiments showed that the transcriptional activity of the receptor was reduced by the simple presence of the RXRα ligand binding domain; it was also unaffected or slightly decreased while exposed to RXRα antagonists, but enhanced in a graded fashion when the receptor was exposed to ligands which normally activate RXRα or the RXRα-Nurr1 heterodimer.

The classic model of transactivation involves an activating ligand stabilizing certain receptor conformations, which then promotes the recruitment of a repertoire of coregulator proteins that enhance gene expression (Nettles and Greene, 2005). To examine whether this mechanism could explain their results, Yu et al. tracked the molecules using a biochemical FRET assay. However, the experiments showed that Nurr1 transactivation does not in fact correlate with a ligand-induced increase in coactivators binding to RXRα; this suggests that another, non-classical process is involved instead.

To further investigate how Nurr1 is activated via RXRα ligands, Yu et al. relied on a technique known as isothermal titration calorimetry to precisely dissect the binding dynamics of these various molecules. The experiments revealed that an increase in Nurr1 transactivation is linked to a weakening of the RXRα-Nurr1 heterodimer. More precisely, the analyses show that the formation of the heterodimer releases energy, and is therefore a more stable, favored state; the binding of the ligands onto RXRα, on the other hand, increases the disorder in the system and makes the formation of the heterodimer more difficult.

Next, Yu et al. delved deeper into how exactly Nurr1 becomes activated after the binding of RXRα ligands onto the RXRα-Nurr1 heterodimers. For this, they used nuclear magnetic resonance, which allows them to observe RXRα and Nurr1 in their various configurations. The data revealed that in the presence of the most effective RXRα ligands, Nurr1 shifts from being part of a heterodimer towards existing on its own. The team further interrogated these results by using size-exclusion chromatography, a ‘molecular sieve’ approach which sorts out molecules based on their size. This showed that the RXRα ligands that are the most effective at activating the orphan receptor favored both Nurr1 existing on its own and four RXRα coming together to form homotetramers. Together, these findings point towards RXRα ligands activating Nurr1 by ejecting it from the heterodimer, and then keeping it on its own by ‘trapping’ RXRα inside oligomers (Figure 1).

**Figure 1.**
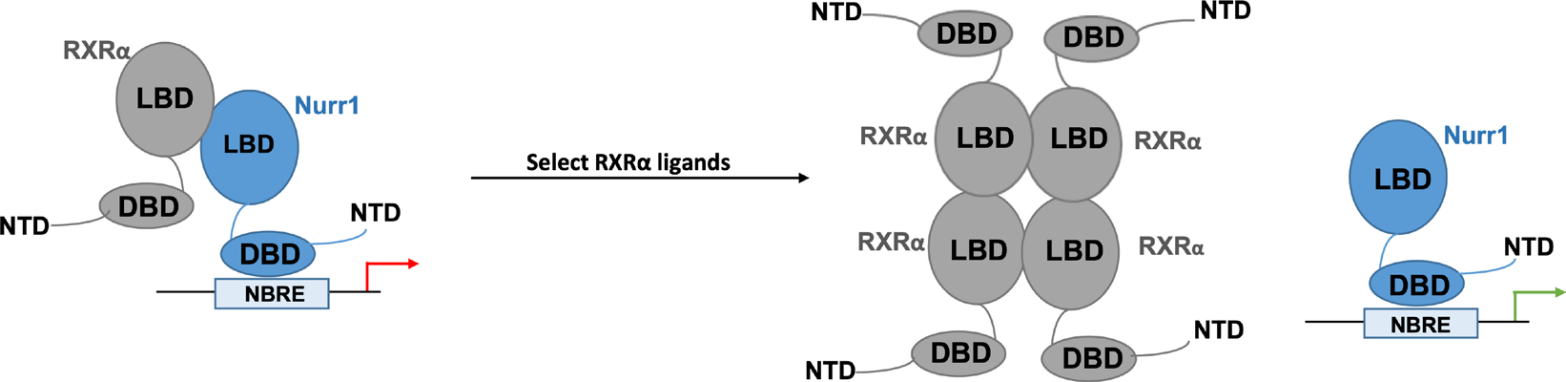
Nurr1 activation may result from selected RXRα ligands disrupting RXRα-Nurr1 heterodimers. When activated, the orphan nuclear receptor Nurr1 (blue) attaches to DNA response elements (known as NBRE) through its DNA-binding domain (DBD) to promote the transcription of genes that help to regulate the activity of dopaminergic neurons. Previous work has shown that the ligand-binding domain (LBD) of Nurr1 has atypical characteristics which point towards the receptor not being directly activated by ligands. Nurr1 can form heterodimers with another nuclear receptor, RXRα, which reduces its transcriptional activity (red arrow). The work by Yu et al. shows that the binding of certain RXRα agonists leads to the activation of Nurr1. They propose a model by which the ligands destabilise the Nurr1-RXRα heterodimer, leading to four RXRα receptors assembling into a tetramer that prevents reassembly with Nurr1, and Nurr1 existing as a monomer with increased transcriptional activity (green arrow).

Taken together, these results reveal an alternative mode of activation for nuclear receptors, one that goes beyond classic regulation mechanisms which require a ligand to occupy the main binding pocket. It is worth noting that the most effective Nurr1 activator was BRF110, an RXRα ligand that has shown therapeutic promise in mouse models of Alzheimer’s and Parkinson’s disease (Spathis et al., 2017). Future work should explore the details of this new mechanism, as well as how to harness it to better investigate and ultimately control the transcriptional activity of Nurr1 and other nuclear receptors that form heterodimers with RXRα.
